# A dimensional approach to affective disorder: The relations between Scl-90 subdimensions and QEEG parameters

**DOI:** 10.3389/fpsyt.2022.651008

**Published:** 2022-08-15

**Authors:** Sermin Kesebir, Ahmet Yosmaoglu, Nevzat Tarhan

**Affiliations:** NPIstanbul Brain Hospital, Üsküdar University, Istanbul, Turkey

**Keywords:** affective disorder, dimensional approach, QEEG, SCL-90, CFC delta beta coupling

## Abstract

**Objectives:**

QEEG reflects neuronal activity directly rather than using indirect parameters, such as blood deoxygenation and glucose utilization, as in fMRI and PET. The correlation between QEEG spectral power density and Symptom Check List-90-R may help identify biomarkers pertaining to brain function, associated with affective disorder symptoms. This study aims at determining whether there is a relation between QEEG spectral power density and Symptom Check List-90-R symptom scores in affective disorders.

**Methods:**

This study evaluates 363 patients who were referred for the initial application and diagnosed with affective disorders according to DSM-V, with QEEG and Scl-90-R. Spectral power density was calculated for the 18 electrodes representing brain regions.

**Results:**

Somatization scores were found to be correlated with Pz and O1 theta, O1 and O2 high beta. Whereas FP1 delta activities were correlated with anxiety, F3, F4, and Pz theta were correlated with obsession scores. Interpersonal sensitivity scores were found to be correlated with F4 delta, P3, T5, P4, T6 alpha and T5, and T6 theta activities. While depression scores were correlated with P3 and T4 delta, as well as T4 theta, there was a correlation between anger and F4, as well as T4 alpha and F8 high beta activities. Paranoia scores are correlated with FP1, F7, T6 and F8 theta, T5 and F8 delta, and O2 high beta activities.

**Conclusions:**

According to our results, anxiety, obsession, interpersonal sensitivity, depression, anger, and paranoia are related to some spectral powers of QEEG. Delta-beta coupling seems to be a neural biomarker for affective dysregulation.

## Introduction

Kraepelin, who suggested the medical disease model for the first time in psychiatry a hundred years ago, included recurrent unipolar depression under the category of bipolar disorder and conceptualized the spectrum disorders (1). As current classification systems are based on cross-sectional diagnosis, they ignore family history, longitudinal course features, and dimensional approach to symptoms. This approach gives precedence to reliability over validity. Descriptive diagnostic systems also disregard neurobiological heterogeneity. However, individual hereditary differences are attributable to differences in neural and physiological function. Many quantitative electroencephalography (QEEG) indicators have the capability to distinguish between psychiatric conditions of affective spectrum (2).

QEEG reflects neuronal activity directly rather than using indirect parameters, such as blood deoxygenation and glucose utilization, as in fMRI and PET. Additionally, QEEG has very high temporal resolution. Cortical discharges of 1.5 mV amplitude are amplified and decomposed with Fourier transformation. Spectral power density is the power EEG waves carry per unit frequency in a defined frequency range. QEEG eliminates the confusion originating from the need to take references. Therefore, this new approach is reported to yield more direct results (3).

This study aims at determining the probable relation between QEEG spectral power density and Symptom Check List-90-R symptom scores of the newcomers diagnosed with affective disorders in psychiatry outpatient clinic.

## Methods

The sample of this cross-sectional study was composed of 816 patients who are drug-free but with psychiatric complaints, applying for the first time in the outpatient clinic of NPIstanbul Brain Hospital, between 2017 and 2018. Patients gave written informed consent in accordance with the Declaration of Helsinki. The Institutional Review Board of Uskudar University approved the study. All data and material archived at our institution according to Information and Consent Form on processing and protection of Personal Data.

QEEG and Symptom Check List-90-R (Scl-90-R) are routine evaluation tools in our NPIstanbul Brain Hospital outpatient clinic. Scl-90-R is present in cross-sectional data with symptom profile. QEEG and Scl-90-R were applied to all consenting participants (n= 702). A total of 528 patients were diagnosed with affective disorders according to DSM-V criteria: depressive disorder, bipolar disorder, and no other specified (NOS). Exclusion criteria were as follows: being younger than 18 and older than 65 years of age (*n* = 35); presence of intellectual disability, dementia, delirium or other amnestic disorders (*n* = 8), and chronic medical diseases (*n* = 122).

All QEEGs were recorded in a quiet, subtly lit room, in sitting position, with eyes closed. Nineteen scalp electrodes were placed according to the 10–20 system. Linked mastoid electrodes (A1–A2) were used for reference. Recording time was 3 min. The data of each subject were averaged across the recording epochs for each electrode and absolute power was computed for the five bands. The article implements 18 electrodes by 128 frequency bins, ranging from 1 Hz to 30 Hz with a resolution of.0078 Hz. We implemented a normative database embedded in the Neuroscan software z-scores which calculates of spectral values. The Z-scores were used to correct individual variation. Spectral power density was calculated for the 18 electrodes representing brain regions (FP1, F3, C3, P3, O1, F7, T3, T5; F4, C4, P4, O2, F8, T4, T6; Fz, Cz, and Pz).

QEEG data were analyzed using Neuroguide Deluxe v.2.5.1 (Applied Neuroscience, Largo, FL). The statistical analyses were performed using the SPSS (Statistical Package for the Social Sciences) 20 software for 363 patients. The Kolmogorov-Smirnov test was used to check for normal distribution. The correlation analysis was performed with Pearson's correlation test according to normal distribution. The false discovery rate (FDR) was computed using methodology described by Benjamini and Hochberg. Significant results were determined based on an FDR-adjusted *p*-value of ≤ 0.05. Regression analysis is applied with ROC curve.

## Results

There are 363 cases, of which 207 are women and 156 are men, with mean age of 29.4 ± 5.1 (Table 1).

**Table 1:** Scl-90-R scores in patient diagnosed with affective disorders.

**Scl-90-R**	**Score (mean ±SD)**
Somatization	13.9 ± 7.2
Anxiety	8.3 ± 5.2
Obsession	9.8 ± 6.3
Phobia	3.2 ± 2.7
Interpersonal sensitivity	7.7 ± 2.1
Depression	14.6 ± 5.9
Anger	14.4 ± 3
Paranoia	12.6 ± 4.1

Somatization scores were found to be correlated with Pz and O1 theta, and O1 and O2 high beta (Table 2); whereas FP1 delta activities were correlated with anxiety, and F3, F4, and Pz theta were correlated with obsession scores. Interpersonal sensitivity scores were found to be correlated with F4 delta, P3, T5, P4, and T6 alpha, as well as T5 and T6 theta activities. While depression scores were correlated with P3 and T4 delta, and T4 theta, there was a correlation between anger and F4 and T4 alpha and F8 high beta activities. Paranoia scores are correlated with FP1, F7, T6 and F8 theta, T5 and F8 delta, and O2 high beta activities.

**Table 2:** Correlations between QEEG spectral power density and psychiatric symptomatology (Scl-90-R) in affective disorders.

	**Frequences**	***r***	***p***	**FDR p**
Somatisation	Pz theta	0.413	0.028	0.048
	O1 theta	0.357	0.031	0.050
	Fz beta	0.223	0.046	NS
	O1 beta	0.261	0.047	NS
	O1 high beta	0.255	0.047	0.052
	O2 beta	0.318	0.030	NS
Anxiety	02 high beta	0.325	0.031	0.055
	FP1 delta	0.452	0.028	0.035
	F4 delta	0.238	0.047	NS
Phobia	Fz delta	0.307	0.045	NS
	O1 high beta	0.283	0.045	NS
	O2 beta	0.357	0.044	NS
Obsession	O2 high beta	0.288	0.045	NS
	F3 theta	0.511	0.007	0.010
	F7 theta	0.345	0.046	NS
	F4 theta	0.489	0.021	0.030
	T4 theta	0.314	0.047	NS
	Cz theta	0.336	0.046	NS
	Pz theta	0.401	0.033	0.050
Interpersonal sensitivity	F4 delta	0.520	0.018	0.031
	Fz delta	0.291	0.045	NS
	C3 alpha	0.383	0.033	0.055
	P3 alpha	0.403	0.033	0.052
	T5 alpha	0.457	0.025	0.033
	C4 alpha	0.313	0.047	NS
	P4 alpha	0.412	0.030	0.050
	T4 alpha	0.297	0.046	NS
	T6 alpha	0.510	0.018	0.030
	Cz alpha	0.253	0.047	NS
	Pz alpha	0.275	0.046	NS
	C3 theta	0.289	0.046	NS
	P3 theta	0.303	0.045	NS
	T5 theta	0.547	0.015	0.028
	C4 theta	0.321	0.044	NS
	P4 theta	0.241	0.047	NS
	T4 theta	0.269	0.047	NS
	T6 theta	0.512	0.018	0.030
	Cz theta	0.245	0.047	NS
Depression	Pz theta	0.256	0.047	NS
	C3 delta	0.347	0.040	NS
	P3 delta	0.495	0.018	0.031
	F8 delta	0.252	0.047	NS
	T4 delta	0.563	0.010	0.022
	F8 theta	0.263	0.046	NS
	T4 theta	0.536	0.014	0.028
	F8 beta	0.301	0.045	NS
	T4 beta	0.283	0.046	NS
	F8 high beta	0.209	0.048	NS
	T4 high beta	0.227	0.048	NS
Anger	F4 alpha	0.378	0.025	0.035
	T4 alpha	0.413	0.018	0.031
	F7 alpha	0.222	0.048	NS
	F7 theta	0.231	0.048	NS
	T5 high beta	0.244	0.048	NS
	Fz high beta	0.237	0.048	NS
	F8 delta	0.326	0.038	NS
	F8 theta	0.328	0.038	NS
	F8 beta	0.247	0.047	NS
	F8 high beta	0.601	0.002	0.010
	FP1 theta	0.532	0.011	0.020
Paranoia	F7 theta	0.551	0.010	0.021
	T6 theta	0.397	0.025	0.030
	T5 delta	0.498	0.015	0.022
	P4 delta	0.317	0.042	NS
	F8 delta	0.404	0.025	0.031
	F8 theta	0.478	0.015	0.028
	FP1 high beta	0.338	0.042	0.057
	O2 high beta	0.479	0.015	0.029

r > 0.2, p < 0.05, FDR adjusted.

**Figure 1:**
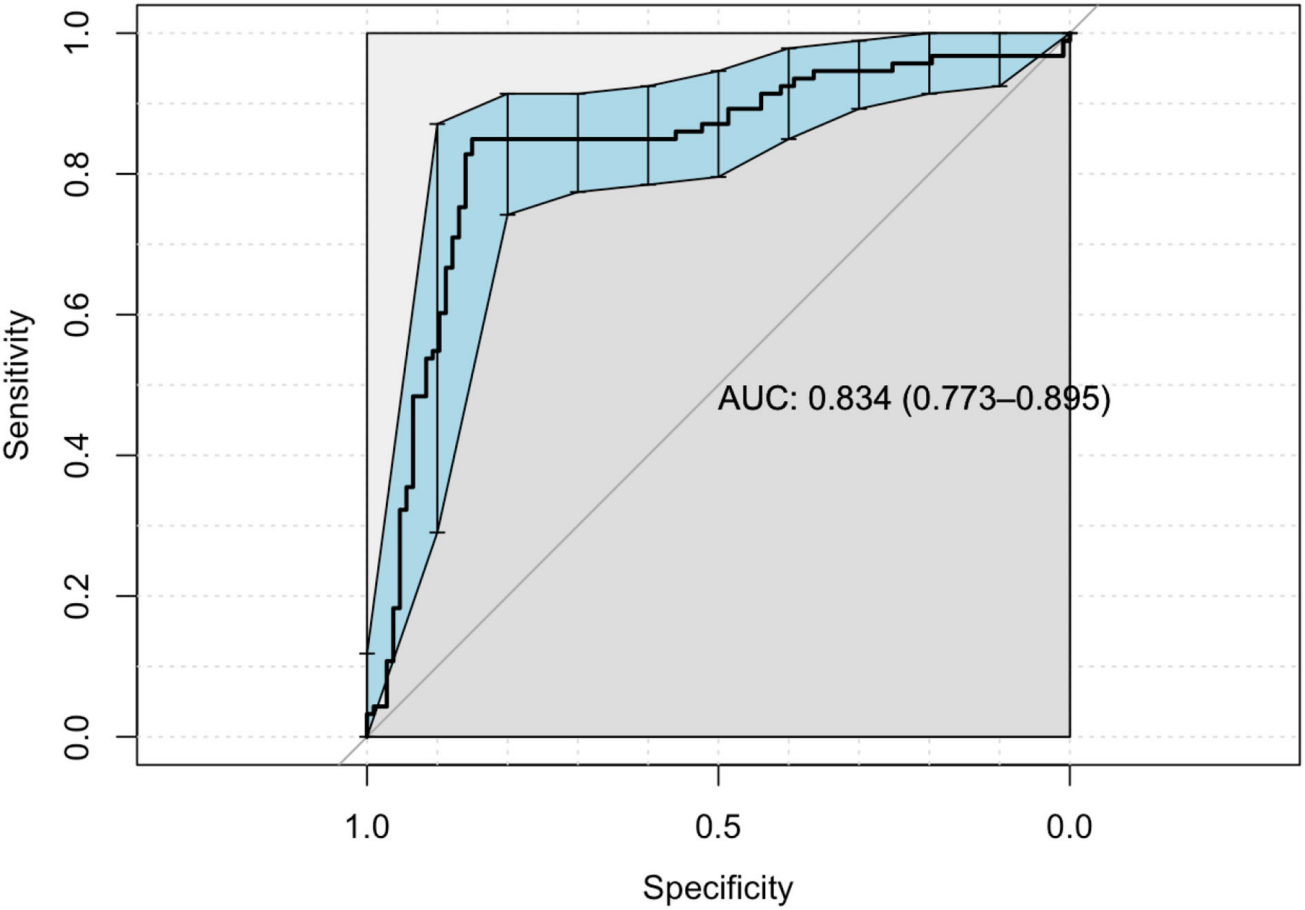
ROC for SCL-90-R.

When regression is implemented to significant correlations, the areas under the ROC curve (AUC) are significant for dimension obsession, interpersonal sensitivity, depression, anger, and paranoia (*R*^2^ = 0.79, *p* < 0.001), (Table 3, Figure 1).

**Table 3:** Regression of correlations between QEEG spectral power density and psychiatric symptomatology (Scl-90-R) in affective disorders.

	**AUC**		**β**	**p**
Obsession	0.653	F3 theta	3.3	<0.01
Interpersonal sensitivity	0.711	T5 theta	3.5	<0.01
Depression	0.770	T4 delta	5.6	<0.001
Anger	0.833	F8 high beta	7.4	<0.001
Paranoia	0.725	F7 theta	6.1	<0.001

## Discussion

This study is the first one to investigate the symptomatology with spectral power density of QEEG in cases with affective disorders. Conventional EEG studies demonstrated EEG anomalies in 20–40% of cases diagnosed with major depressive disorder (MDD) (4). In a study on first manic episode cases, we had showed this rate to be 28.7% (5).

A slowed posterior dominant rhythm is characteristic for a major depressive disorder (3). Specificity and sensitivity of QEEG changes were reported as 65–93% and 72–88%, respectively. In this study, a correlation was found between depression scores and P3, and T4 delta and T4 theta power. At this juncture, the important question is whether depression cases with severe cognitive cases, also known as pseudodementia, will exhibit similar findings with cases of dementia. Although some studies tend to approve this (6), we think the slowing in MDD is functional and will be normalized with recovery. On the other hand, the mean age of our subjects is rather low for dementia. A similar discussion on differential diagnosis must be made with schizophrenia about motivational and attentional factors. Hughes and John (4) suggested that an increased frontal theta and alpha activity, which is present in our cases, will differentiate depression cases from schizophrenia, in which opposite findings exist (4). Delta, theta, and alpha slow-wave activities are correlated with immobilization in animal models, whereas beta power shows negative correlation (7). Unipolar and bipolar depressions are different regarding QEEG changes (3). Lieber and Newbury (8) reported two kinds of depression findings. QEEG data in their study on 216 in-patients (8): the first group demonstrated an increase in beta and/or slow wave activity, whereas the second group had an increase in slow wave activity. We interpret the QEEG findings of the first group as cases of bipolar depression with mixed features. This distinction will become clearer toward beta power, while alpha activity is normalized under antidepressive medication.

A correlation among anger scores, F4, and T4 alpha, as well as F8 high beta power, was found in this study. Alpha activity was interpreted in favor of bipolar depression when found together with beta activity, and in favor of mania, when it is correlated with beta power alone. As a matter of fact, frequency diversity is higher in the manic period than in remission and depressive period, while increased beta activity is frequently observed (9, 10).

Paranoia scores were correlated with FP1, F7, T6, and F8 theta; T5 and F8 delta, O2 beta powers. No study on QEEG in affective disorders with psychotic features was found in the literature. Also, the frontal and occipitoparietal alpha activity that distinguished between psychotic and non-psychotic manic cases in our previous study was not found in consequential remission period in this study done on acutely presenting cases. In their hypothesis testing study, Buckley and Miller (11) reported that frontal delta, alpha, and occipitoparietal beta activities do not predict transition to psychosis. Nuwer et al. (12) and Hughes and John (4) thought that QEEG does not have consistency regarding phenomenological findings and subtypes and does not have a value in predicting treatment response in schizophrenia. We agree with these expert opinions and to take a step further, we postulate that this condition itself is a contribution to differential diagnosis in cases that are hard to differentiate from affective disorders. EEG abnormality, which was reported at 5–80%, is usually seen in the form of reduced alpha power in the frontal lobe and is normalized with antipsychotic treatment (13).

Suffin and Emory (14) studied 54 unmedicated patients diagnosed with affective disorders and 46 with ADHD. They found partial increased of alpha of both groups and increased theta in the other, and they reported a better response to stimulants in cases with increase frontal theta activity. While they stress the importance of this finding in cases unresponsive to antidepressants and anticonvulsants, Lee et al. (15) reported an association between theta activity and suicidal ideation.

The bodies of evidence summarized up to this point regarding affective disorders and QEEG are necessary but not adequate, especially for subgroups.

Interpersonal sensitivity is a usual finding in affective disorders, especially in bipolar affective disorder type II (16). This study revealed a correlation among interpersonal sensitivity scores and F4 delta, P3, T5, P4, T6 alpha, and T5 and T6 theta activities. In a study on depressive female cases, Hunter et al. (17) found a correlation between prefrontal theta cordance and T5, as well as between T6 theta activities and interpersonal sensitivity, and reported the antidepressant response as remission.

There is a correlation between somatization scores and FP1 delta, F4 and F8 delta and theta, and T4, T6, and O2 theta activities. A predisposition defined as ‘dissociative mental state' in the literature is reported to coexist with posterior temporal and parietooccipital EEG abnormalities in 58% of the cases (18). The primary abnormality is increased theta power, predominantly in temporal electrodes in 23 of 29 cases in another study (19). The phenomenologies of these cases were conversion and depersonalization. These findings do not contradict ours.

Obsession scores are found to be correlated with F3, F4, and Pz theta activities. Zielinska et al. (20) mentioned the increased parietal alpha and frontal beta activities in obsessive compulsive disorder (OCD). Prichep et al. (21) defined to groups of unmedicated OCD cases, in which frontal alpha and frontotemporal theta activities are increased, consistent with pathophysiologically and clinically described subtypes. As a matter of fact, 82% of cases showing increased alpha activity respond to SSRI's. An important question is whether this SSRI response is specific or associated with comorbid depression. On the other hand, it must not be forgotten that our subjects were diagnosed with affective disorders. Hansen et al. (22) studied 20 patients with OCD in a study independent from Prichep et al.'s, and they predicted that 18 cases will respond to paroxetine, in line with Prichep et al.'s findings. Seventeen of the cases were treated blindly responded to paroxetine. At this point, we propose that in affective disorders with obsessive compulsive features, the obsessive-compulsive symptoms will not respond well to SSRIs.

We found correlations between anxiety scores and FP1 delta power. Studies comparing panic disorder cases with healthy controls (23, 24) point to frontal alpha activity with 71% sensitivity and 84% specificity. De Carvalho et al. (25) demonstrated the increase of frontal beta power as a differentiation factor in panic disorder with agoraphobia. They emphasize anticipatory anxiety and hypervigilance associated with this finding.

To summarize our findings up to this point, we suggest that beta power, which was associated with bipolar depression, mania, schizophrenia-like psychoses, OCD, phobia, and panic disorder with anxiety in the literature, which we accordingly found to be associated with depression, anger, paranoia, obsession, and phobia scores, is characterized with anxiety accompanied by hypervigilance. This mood reactivity was linked with irritability (26). On the other hand, delta power is found to be associated with anxiety scores as well. At this point, we propose a continuity between slow-wave activity and fast-wave activity, such as the cross-frequency coupling (CFC) between delta and beta power. CFC between different neural oscillations is a key functionality, which the brain coordinates complex cortical computations (27). It can be thought of as a kind of stress response that is aberrant and regulatory. An increase power of delta reflects increased activity of subcortical affective processes, e.g., anhedonia, reward dependency, and impulsivity, whereas an increase power of beta reflects an effort for cortical regulation of negative emotion with cognitive process, e.g., working memory and sustainable attention.

Relationships between slow and fast wave frequency bands are considered to be of interest of dispositional affective traits as a continuity between depression and mania proposed first by Areteus, such as mania is a severer form of depression and anxiety, (1) accompanied by hypervigilance and vegetative symptoms, and is characterized by retardation. Indeed, we have shown that phase amplitude coupling (PAC) delta beta is related with mixed features and response to treatment in bipolar II depression (28).

These results are discussed with a focus on the correlations of spectral power density of brain regions and affective temperament in predicting risk, resistance, and resilience for bipolar disorder in our previous study (29). Affective temperament is situated in the mildest end of the bipolar spectrum. Affective temperament is a suggested endophenotype for BD as well (30). In the mentioned study, F4 and T4 delta activities were similar between patients and their relatives, whereas Pz alpha activity was similar in relatives and unrelated healthy subjects. Cyclothymic and hyperthymic temperament scores were found to be similar between patients and their relatives and higher than unrelated healthy controls. F7 beta and F7-O2 high-beta power were correlated with hyperthymic and irritable temperament, respectively in patients who are bipolar. T3-F4-T4 delta powers were correlated with cyclothymic temperament in patients and healthy relatives. An inverse correlation was found between Pz alpha power and hyperthymic temperament in healthy relatives and unrelated healthy subjects. According to this results, medial temporal network seems to be associated with the heritability of bipolar disorder. However, left dorsolateral prefrontal beta and high beta activity may be a neural marker of resistance for the disorder in addition to right occipital high beta power. The inverse relation between hyperthymic temperament scores and Pz alpha power can be associated with resilience in healthy relatives and unrelated healthy subjects.

In the mentioned study, all patients who are euthymic bipolar included in the study were on lithium prophylaxis. The aim of giving a uniform treatment was to standardize the prophylactic treatment. Whilst lithium normalizes beta, delta, and theta activities, treatment response is most closely associated with basal delta activity (31, 32). In light of our findings, the association between the delta activities of healthy first-degree relatives and bipolar patients in ongoing remission with lithium, diverting from unrelated healthy controls, seems meaningful in this aspect. In favor of our hypothesis, it was shown that dominant beta activity can be converted to alpha activity with diazepam in mice (33). A better motor performance was achieved with bromozepam in the presence of increased beta activity, (34) and it is shown to have a neuromodulatory effect on procedural learning (35). The same mechanism may be antimanic and mood stabilizing effects of anticonvulsants.

### Limitations and suggestions

Methodological differences, such as recording different brain regions, using different hardware configurations, complicate the replication of study findings and diminish the consistency of the normative database, in which an abnormal EEG rate of 12% is reported among healthy subjects (36). While type 1 error concluded with 84% sensitivity, specificity is determined as 91%. Positive predictive value was calculated as 98% and negative predictive value as 53%. Future normative databases must be built considering genetic, geographical, climatic, social, and cultural similarities and differences. Another limitation is the effect of medication on the recording. A major strength of the manuscript is its large sample size, including people with a diagnosed affective disorder and who were not taking medication at the time of the testing. Having all the participants completed both QEEG and SCL-90-R makes the dataset a valuable asset. Comparing of subgroups of affective disorders or machine learning could be the subject of another study.

## Conclusion

In conclusion, a considerable number of authors have the opinion that QEEG findings are not clearly associated with specific diagnostic categories. At this point, the absence of evidence must be carefully distinguished from evidence of absence. However, a dimensional approach will yield more consistent results than categorical diagnosis. In this study we set forth relations with high internal and external consistencies about affective disorders. CFC delta-beta coupling seems to be a neural biomarker for affective dysregulation.

## Data availability statement

The raw data supporting the conclusions of this article will be made available by the authors, without undue reservation.

## Ethics statement

The studies involving human participants were reviewed and approved by the Institutional Review Board of Uskudar University. The patients/participants provided their written informed consent to participate in this study.

## Author contributions

SK conceived and designed the experiments, performed the experiments, and analyzed and interpreted the data with AM. SK wrote the paper. All authors contributed to the article and approved the submitted version.

## Conflict of interest

The authors declare that the research was conducted in the absence of any commercial or financial relationships that could be construed as a potential conflict of interest.

## Publisher's note

All claims expressed in this article are solely those of the authors and do not necessarily represent those of their affiliated organizations, or those of the publisher, the editors and the reviewers. Any product that may be evaluated in this article, or claim that may be made by its manufacturer, is not guaranteed or endorsed by the publisher.
